# Drowning

**Published:** 1873-06

**Authors:** 


### DROWNING.

Some persons have been brought to life as late as three hours after having been taken from the water, and yet cases are occurring where life failed to be restored, although the body was recovered within twenty minutes after falling into the water, doubtless from the fact that none were present who knew how to treat a drowned person. A drowned person dies because the lungs are so full of water that no air can get in Place the person so that the forehead shall rest on the arm, to keep the nose from touching the ground or bed; get astride the body, and with both hands under the middle raise it up, making a declivity to favor the water running from the bottom of the lungs, which are now highest, towards the mouth, which is the lowest, taking care that the mouth be kept open by drawing the tongue forward, hoisting and lowering the body quickly all the time. If there is no appearance of life within five minutes, the Belgian experiment should be tried. Place an iron plate heated to a white heat for an instant over the pit ot the stomach, which is just below the breast bone ; a person came to life after this treatment who had been seemingly dead for three hours; the plate should be about five inches across ; it is a desperate remedy, but the case is desperate. An iron red hot causes more suffering than if white heated. While the iron is in preparation, do all possible to keep the body warm with hot baths to the feet, under the arms and over the stomach, keeping several hands busy in rubbing the skin whereever it is practicable. In rescuing drowning persons, approach them from behind, and hoist them up by the hair of the head.

A little boy fell into a canal ten feet deep, and soon sank out of sight, but one of his companions, only eleven years old, promptly dived after him; after several efforts he grasped the lad around the neck, raised him to the top of the water and pulled him on shore, apparently dead. Not satisfied with what he had done, he began to try to bring him to life by placing him on his stomach and rolling him gently but quickly from side to side; this caused a free vomiting of water ; the air took its place, and the boy was saved. The ordinary directions about resuscitating drowned persons are obscure and complicated, but every one should acquaint himself with this method, and which should always be carried out as promptly as possible.

